# Comparisons Between Bacterial Communities in Mucosa in Patients With Gastric Antrum Ulcer and a Duodenal Ulcer

**DOI:** 10.3389/fcimb.2018.00126

**Published:** 2018-05-08

**Authors:** Xia Chen, Chenmei Xia, Qianqian Li, Linxiao Jin, Liyun Zheng, Zhongbiao Wu

**Affiliations:** ^1^Department of Gastroenterology, The First People's Hospital of Wenling, Taizhou, China; ^2^Department of Research Service, Zhiyuan Inspection Medical Institute, Hangzhou, China; ^3^Department of Urology, The First People's Hospital of Wenling, Taizhou, China

**Keywords:** microbial community structure, *Helicobacter*, gastric antrum ulcer, duodenal ulcer, 16S rRNA

## Abstract

**Objective:** To identify and compare the bacterial community profile of mucosal tissues from a gastric antrum ulcer and a duodenal ulcer in *Helicobacter pylori* (*Hp*) positive dyspeptic patients.

**Methods:** Genomic DNA was extracted from the mucosal tissues obtained from 18 patients diagnosed with gastric antrum or duodenal ulcers. A library was constructed using 16S rRNA gene amplification, and Miseq high-throughput sequencing was used to analyse the amplified products. Bioinformatics methods, including operational taxonomic units (OTUs), hierarchical clustering, and a diversity analysis, were performed to investigate and characterize the community composition.

**Results:** The proportion of *Helicobacter* in the mucosa of patients with a gastric antrum ulcer was significantly higher than that of patients with a duodenal ulcer. However, the diversity of the bacterial community in the gastric antrum ulcer mucosa was significantly lower compared with the mucosa of the duodenal ulcer. There were significant differences in microbial community structure between the gastric antrum ulcer and the duodenal ulcer. Notably, *Helicobacter, Prevotella, Neisseria*, and *Streptococcus* were also predominant genera in the bacterial community of the duodenal ulcer mucosa, and they outnumbered those species in gastric antrum ulcer mucosa.

**Conclusion:** The bacterial community composition and the corresponding abundance differ between the mucosal tissues of *Hp* positive gastric antrum ulcer and duodenal ulcer patients. Additionally, the bacterial community diversity in the mucosal tissues from gastric duodenal ulcer patients is higher than that from gastric antrum ulcer patients, and *Helicobacter* is not the absolutely predominant genus.

## Introduction

Gastric ulcers and duodenal ulcers are two common peptic diseases seen in the clinic. A *Helicobacter pylori* (*H. pylori*) infection is an important causal factor (Graham and Shiotani, 2008) associated with more than 80% of peptic ulcers (Alam et al., 2014). Previous studies have reported that the occurrence of ulcers leads to alterations in the stomach microbiota (Li et al., 1995; Zhang et al., 1996), by aerobic bacteria, anaerobic bacteria, fungal isolation, and culture. However, this method has its limitations; it could not fully demonstrate the change of stomach microbiota in patients with peptic ulcers.

Recent years have witnessed the rapid development of high-throughput sequencing technology, especially the wide use of the bacterial 16S rRNA-encoding gene sequence analysis, which has made a significant improvement in the identification of bacterial lineages and their relative abundance in the gastric microbial community. For example, in non-*Hp* infected patients, Zilberstein et al. (2007) found predominantly acid resistant species—*Veillonella* sp. *Lactobacillus* sp. and *Clostridium* sp.—in the digestive tract microbiota, while in *H. pylori*-infected patients, some non- *Hp* bacterial genera such as *Streptococcus, Neisseria, Staphylococcus*, and *Roche* were also identified in gastric biopsy tissues (Hu et al., 2012). Nevertheless, *Hp* infection is a well-acknowledged causative agent of peptic ulcers (Groenen et al., 2009), and there have been few reports about the microbial structure of different ulcer types, such as gastric ulcers and duodenal ulcers.

The mechanisms of ulcer formation are diverse and include excessive gastric acid secretion and the use of non-steroidal anti-inflammatory drugs (NSAIDs), and these mechanisms are secondary to those in Crohn's disease and tuberculosis (Yuan and Tang, 2008). In recent years, the incidence of non- *Hp* non-NSAIDs-related ulcers has been on the rise (Hou and Zhang, 2015). From a microbial ecology viewpoint, the number of *Hp* that have colonized is not the only reason for a peptic ulcer. Other non- *Hp* bacteria may participate in the development of two common ulcers. Additionally, few studies have paid attention to the tissue structure of gastric and duodenal ulcers. The relevance of the *H. pylori* content and the proportion of other bacteria to ulcer formation and other issues also urgently require resolution.

In this study, to compare the bacterial community profile of the mucosa of a gastric antrum ulcer and a duodenal ulcer, we utilized the Miseq high-throughput sequencing technique and analysis to identify and compare the structural, biological diversity and abundance of the mucosal microbiota of 18 patients with *H. pylori*-positive gastric antrum and duodenal ulcers. The composition of the mucosal microbiota from the different ulcer types can provide a theoretical basis to understand the relevance of the mucosal flora to ulcer disease.

## Materials and methods

### Study population

Gastric and duodenal biopsy samples were obtained between 2016 and 2017 from patients referred for an endoscopy examination at the First People's Hospital of Wenling. Biopsy samples were taken from the antrum and the body of the stomach for each patient. This study was approved by the First People's Hospital of Wenling Medical Ethics Committee, and written consent was obtained from the patients before they were included in the study.

All patients were outpatients admitted in the First People's Hospital of Wenling diagnosed with gastric and duodenal ulcers. Each patient received a ^13^C urea breath test (^13^C using a 75 mg dose) before endoscopy, and the test results were positive. All patients never had *Hp* eradication before gastroscopy. And we surveyed the patients' information, such as histopathological data, diet habits, economic status, and family history.

### Specimen collection and processing

Each patient underwent endoscopic mucosal tissue sampling, and two mucosal tissue specimens were collected in parallel at the same biopsy site. Specifically, 3–5 cm of tissue around the gastric antrum ulcer surface was collected from gastric antrum ulcer patients, and tissue around the duodenal ulcer surface was collected for duodenal ulcer patients. For each patient, one of the collected mucosal tissue samples was placed in an *Hp* isolation culture tube containing a brain-heart infusion and the other was in a sample tube containing DNA preservation solution.

### Bacterial growth and identification

After the sample was received, the tissue was first placed at room temperature, and the gastric mucosal tissue was sucked into a sander with a Pasteur pipette and then sufficiently ground to homogenize it; it was then inoculated on a 5% defibrinated sheep blood agar plate and evenly spread. The inoculated plate was placed in a 37°C three-gas incubator (5% oxygen, 10% carbon dioxide, 85% nitrogen), cultured for 3–11 days, and the growth was observed. Suspected colonies were smeared for microscopic observation of bacterial morphology consistent with *Hp*, and strains that were urease, oxidase, and catalase positive were classified as *Hp* positive, otherwise they were classified as *Hp* negative. Gastric antrum ulcer *H. pylori* positive patients were designated group A (antrum), and duodenal ulcer *Hp* positive patients were designated group B (duodenal).

### DNA isolation, library preparation and sequencing

Genomic DNA from all the mucosal flora samples was extracted using an Invitrogen Purelink Genomic DNA kit (Life Technologies, Carlsbad, CA, USA). The operation was carried out strictly according to the kit instructions. The qualitative and quantitative detection of nucleic acids was accomplished by 1% agarose gel electrophoresis and a Qubit2.0 concentration analyser.

A specific primer with a “sequencing linker” was synthesized for the 16S rRNA V3 and V4 regions of mucosal flora samples. The first step PCR primer sequence was 356F: 5′-TCGTCGGCAGCGTCAGATGTGTATAAGAGACAGCCTACGGGNGGCWGCAG-3′; 806R: 5′-GTCTCGTGGGCTCGGAGATGTGTATAAGAGACAGGGACTACHVGGGTWTCTAAT-3′. The PCR reaction system contained 2 × HiFi buffer 10 μL, forward primer 5 μmol/L, reverse primer 5 μmol/L, DNA 10 ng, plus ddH_2_O to the total volume of 20 μL. PCR reaction conditions: 95°C, 3 min, 25 cycles including 95°C 30 s, 60°C 30 s, 72°C 30 s; 72°C 5 min.

After PCR amplification, the PCR products were purified with Backman magnetic beads. Briefly, 20 μL of the PCR reaction product was mixed with 16 μL of magnetic beads to adsorb the DNA, and then washed with 80% ethanol twice and eluted with 40 μL of ddH_2_O to obtain the purified PCR product. Then, the second step PCR reaction system contained 2 × HiFi buffer 10 μL, forward primer 10 μmol/L, reverse primer 10 μmol/L, first PCR Purify the product template 2 μL, plus ddH2O to a total volume of 20 μL. The PCR reaction conditions were 95°C, 3 min, 12 cycles including 95°C 30 s, 60°C 30 s, 72°C 30 s; 72°C 5 min. After completion of the PCR reaction, purification was accomplished according to the previously mentioned magnetic beads purification protocol, and finally, 30 μL of ddH_2_O was eluted to obtain a DNA library of mucosal samples.

Quality control of all the libraries was conducted using an Agilent 2100 Bioanalyzer analyser and a Qubit 2.0 concentration analyser. The library that passed the qualification was sequenced using an Illumina MiSeq sequencer.

### Analysis of 16S rRNA gene sequences

The original image data of the sequencing results were analyzed by the CASAVA software (v1.8.2), and the sequencing data were obtained after a preliminary quality analysis. Pandaseq (v2.7) was used to compare every two sequences and assemble them according to the end of the overlapping area. Using Trimmomatic (v0.30), the primers and linker sequences, and bases with a mass <20 at both ends as well as sequences with lengths <400 bp were also removed. Using usearch (v8.0), the remaining sequences were compared with those in the database, and the chimeric sequences were removed to obtain the final validated data. Operational Taxonomic Units (OTUs) are clustered sequences into bins, clustering was performed by the UCLUST method with the parameter similarity set at 97%, and an OTU list and OTU representative sequences were obtained. The sequences were randomized using the Qiime platform (v1.7), and dilution curves were constructed using the number of sample sequences and the number of OTU they could represent. According to the results of the OTU clustering, ACE, Chao1, Shannon, and Simpson were used to analyse the abundance and diversity of the mucosal samples. Using the Qiime platform (v1.7), a principal component analysis was performed based on the UniFrac distance.

### Statistical analysis

Statistical analyses were performed using SPSS19.0 software. The comparison of the continuity variables was analyzed using a *t*-test, and the classification of the data was analyzed using the chi-square test or Fisher's exact test. A *P* value ≤ 0.05 was considered as statistically significant.

## Results

### Patient clinical data

In this study, 20 cases were enrolled in the study group, including 10 cases of gastric antrum ulcer and 10 cases of duodenal ulcer. The age range of the patients was 33–67 years, with an average age of 45.3 years and a male to female ratio of 1.857: 1. After *Hp* isolation and culture, one case of gastric antrum ulcer and one case of duodenal ulcer were determined to be *Hp* negative and removed. Thus, a total of 9 *Hp* positive cases remained for the gastric antrum ulcer group. The average age of the patients was 48.4 years, and the male to female ratio was 1.25: 1. For the duodenal ulcer *Hp* positive group of nine patients, the average age was 41 years and the male to female ratio was 2: 1. There was no significant difference between the two groups for age (*p* > 0.05, *T-*test) or sex ratio (*p* > 0.05, *x*^2^ test). There was also no significant difference in histopathological data, diet habits, economic status, and so on between two groups (Table 1).

**Table 1:** The patients' information.

**Groups**	**Sex**	**Age**	**Diet habits**				**Economic status**	**Family history**		**Histopathological data**		
			**Wine**	**Salty**	**Vegan**	**Spicy**		**Stomach cancer**	**Hypertension**	**Ulcer size(cm)**	**Lymphoid hyperplasia**	**Activity**
A1	male	42	U	S	No	Much	Middle	No	Yes	1.0*1.5	No	Yes
A2	male	67	U	S	No	Little	Middle	No	No	0.5*0.5	No	Yes
A3	male	51	S	U	No	Much	Middle	No	No	0.3*0.7	No	Yes
A4	male	40	No	No	No	Little	High	No	No	0.3*0.3	Yes	No
A5	female	60	S	No	No	Little	Middle	No	No	0.3*0.3	Yes	Yes
A6	female	36	S	S	No	Little	Middle	No	Yes	0.3*0.5	No	Yes
A7	male	54	No	S	No	Much	Middle	No	No	1.0*0.5	No	Yes
A8	female	46	No	S	No	No	High	No	No	0.3*0.4	No	Yes
A9	female	40	No	S	No	Little	Low	No	No	0.6*1.5	No	Yes
B1	male	35	No	S	No	Little	High	No	No	0.5*0.6	Yes	No
B2	female	39	S	U	No	Much	Low	No	Yes	0.3*0.4	No	No
B3	male	42	U	S	No	Little	Middle	No	No	0.3*0.5	No	No
B4	male	37	U	S	No	Little	High	No	Yes	0.5*0.4	No	Yes
B5	female	52	No	S	No	Little	Low	No	No	0.5*0.5	No	Yes
B6	male	42	S	S	No	Little	High	No	No	0.4*0.5	Yes	No
B7	female	33	U	No	No	Little	High	No	Yes	0.4*0.5	No	Yes
B8	male	46	No	U	No	Little	Middle	Yes	No	0.3*0.6	No	Yes
B9	male	43	U	U	No	Much	Low	No	Yes	0.6*1.2	No	No

“*S” represents “sometimes”, “U” represents “usually.”*

### Diversity analysis

**Figure 1:**
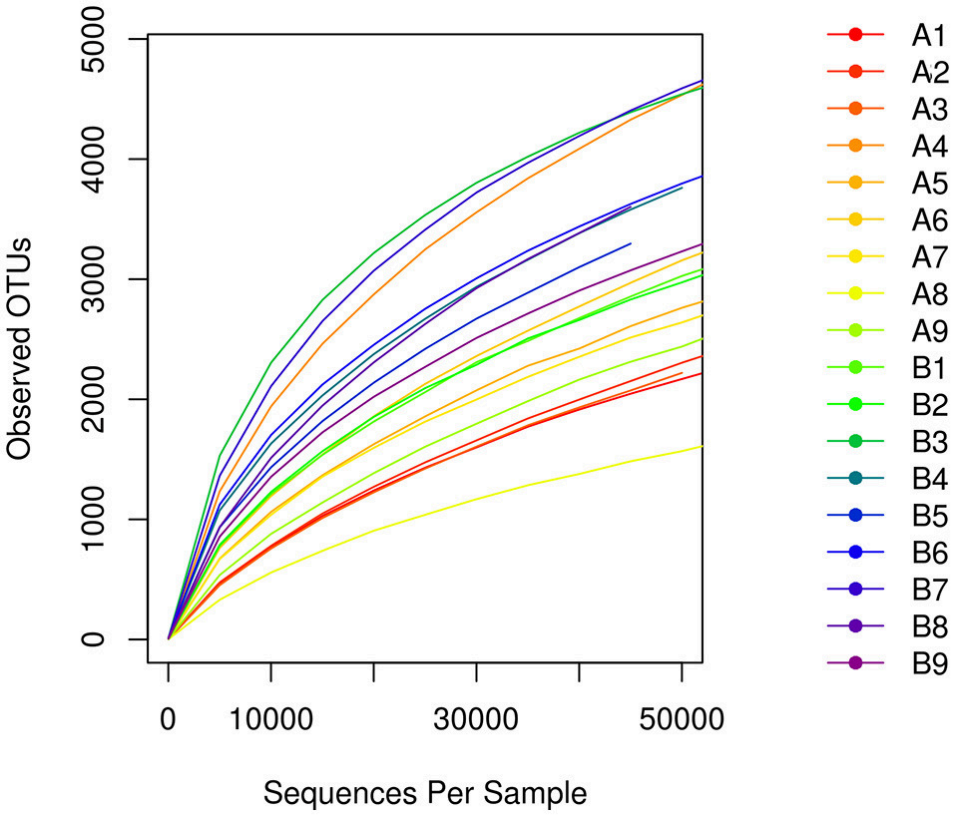
The number of OTUs of the dilution curve increases with the number of sequences.

A total of 1,564,734 primitive sequences were obtained by high-throughput sequencing of the 16S rRNA gene of the mucosal samples using a two-step PCR amplification method. Of these sequences, 1,520,613 sequences were effective, with a coverage index of 97.18%. The sequences were sorted by the 97% similarity of the OTUs, resulting in a total of 32513 OTUs. The number of OTUs was 21578 in the *Hp* positive gastric antrum ulcer patients (group A) and 24226 in the *Hp* positive duodenal ulcer patients (group B) (Table 2). As shown in the sample dilution curve in Figure 1, the number of OTU in the dilution curve increases with the number of sequences without reaching a plateau but tends to reach the platform stage. This indicates that sequencing quantity for each sample was sufficient to characterize the composition of the sample flora. The bacterial diversity in mucosa of duodenal ulcer patients was significantly higher than that of the gastric antrum ulcer patients (Figure 1).

**Table 2:** Bacterial community α-diversity indexes for *Hp* positive gastric ulcer types.

**Group**	**OTUs**	**ACE**	**Chao1**	**Shannon**	**Simpson**	**Coverage**
Antrum	21578	10224.67	9518.19	3.81	0.51	0.97
Duodenal	24226	10349.09	9561.53	7.61	0.91	0.96

*OTUs, Operational Taxonomic Units*.

### Bacterial community composition in a *Hp* positive gastric antrum ulcer vs. a duodenal ulcer

**Figure 2:**
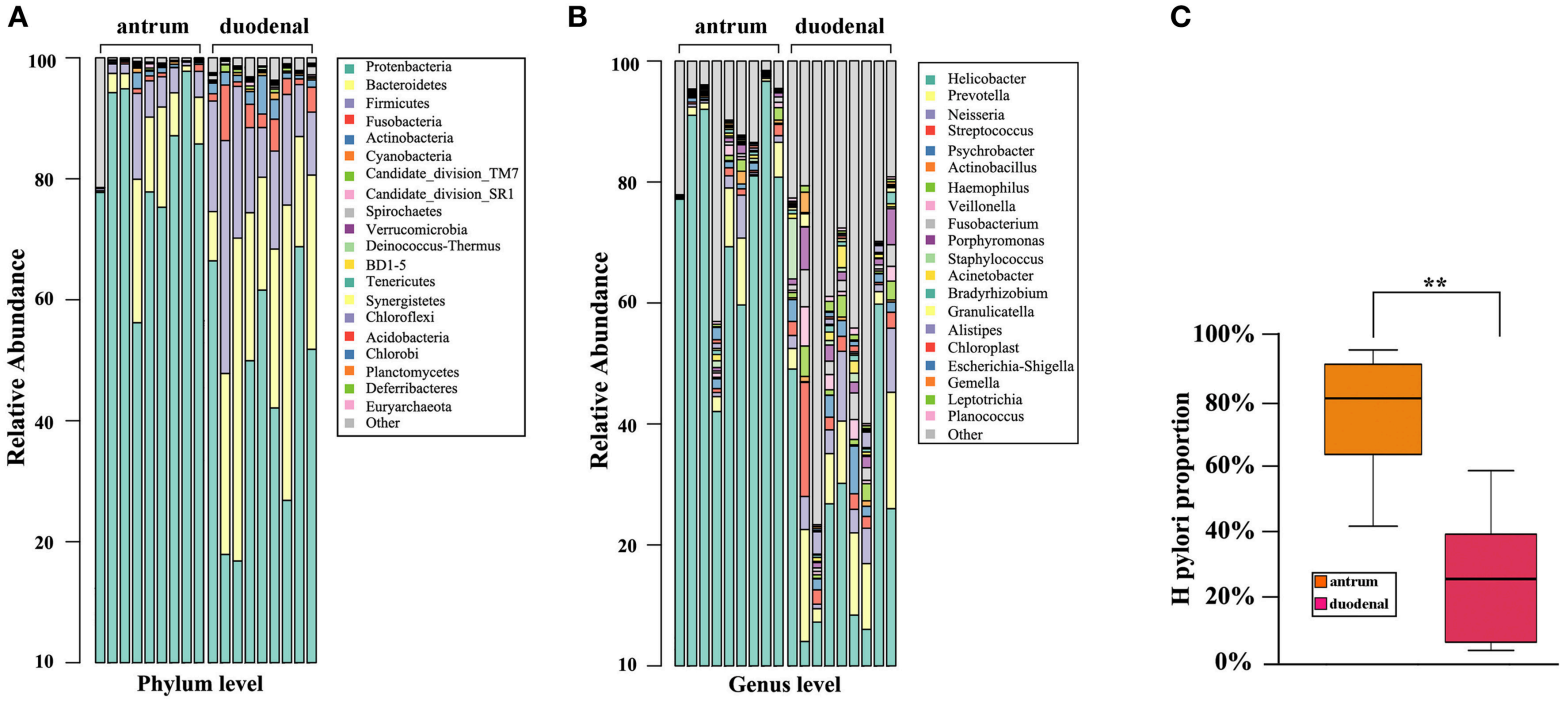
Bacterial community composition for the *Hp* positive gastric antrum and duodenal ulcer types identified at the **(A)** phylum, **(B)** genus levels, and **(C)** groups. ^**^Statistical significant difference in the two groups.

As shown in Figure 2A, the predominant bacterial phyla in the two ulcer types were *Proteobacteria, Bacteroidetes*, and *Firmicutes*. It was noteworthy that *Proteobacteria* accounted for the major proportion in the *Hp* positive gastric antrum ulcers, whereas *Bacteroidetes* and *Firmicutes* accounted for the major proportion in the *Hp* positive duodenal ulcers, and the difference was statistically significant (*p* < 0.05, *x*^2^ test).

The bacterial community composition at the genus level in the two ulcer types is shown in Figure 2B. Overall, the proportion of either the predominant or unknown bacteria was significantly higher for the *Hp* positive duodenal antrum ulcer than that for the *Hp* positive gastric ulcer. *Helicobacter* accounted for the major proportion in the *Hp* positive gastric antrum ulcer, with an over 40% ratio. However, *Helicobacter* was not the predominant bacterial genus in the *Hp* positive duodenal ulcer, only present in less than a 10% proportion in some samples. Notably, bacterial genera including *Prevotella* (9.83%), *Neisseria* (5.03%), *Streptococcus* (3.96%), *Veillonella* (1.92%), *Streptococcus* (3.96%), and *Porphyromonas* (2.62%) were also present in the *Hp* positive duodenal ulcer. Furthermore, the genera *Psychromonas, Frankia, Prevotella*, and *Porphyromonas* were identified and significantly distinguished the *Hp* positive duodenal ulcer samples (*p* < 0.05, *x*^2^ test).

We then determined the proportion of colonization of *Hp* in the *Hp* positive gastric antrum and the duodenal ulcers. As shown in Figure 2C, the average proportion of *Hp* in the gastric antrum ulcer samples was 76.44%, and it was 24% in the duodenal ulcer samples. The difference was statistically significant (*p* < 0.01, *x*^2^ test).

### Bacterial community diversity in the *Hp* positive gastric antrum and the duodenal ulcer

**Figure 3:**
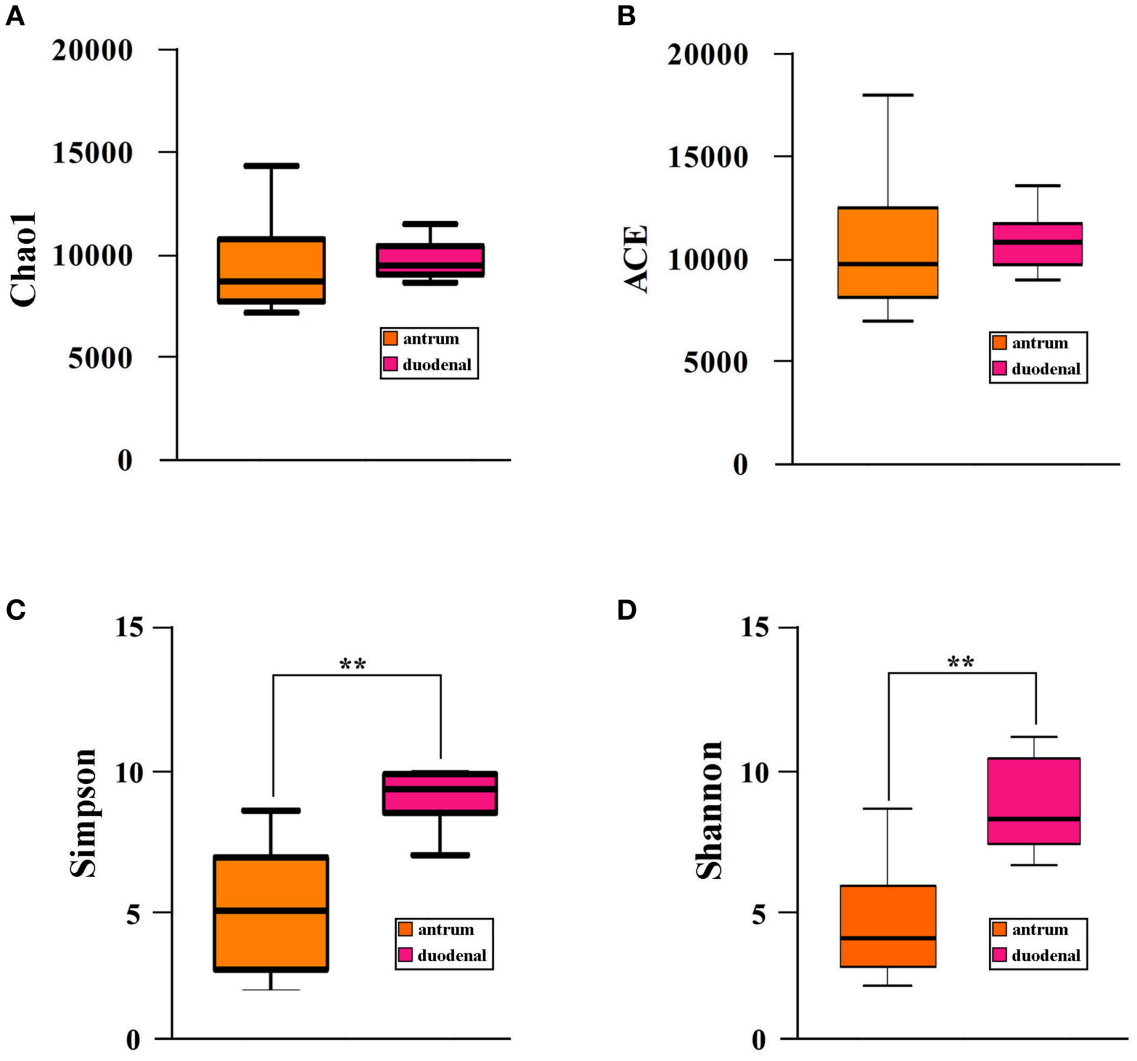
Bacterial community α-diversity **(A)** Chao1, **(B)** ACE, **(C)** Simpson, and **(D)** Shannon, indexes for the *Hp* positive gastric antrum and duodenal ulcers. ^**^Statistical significant difference in the two groups.

We investigated the bacterial community diversity of the two *Hp* positive ulcer types by first calculating the four α-diversity indexes, including the Chao1, Shannon, Simpson, and ACE indexes. For the *Hp* positive gastric antrum ulcers, the four indexes were 9518.19, 3.81, 0.51, and 10224.67 (Table 2). For the *Hp* positive gastric duodenal ulcer, the four indexes were 9561.53, 7.61, 0.91, and 10349.09 (Table 2). The ACE and Chao1 indexes represent the bacterial community richness in a single sample. Clearly, there were no significant differences in ACE and Chao 1 for the two ulcer types (*p* > 0.05, *t*-test, Figures 2A,B), indicating no difference in the total number of identified bacterial species for the two ulcer types. For the Shannon and Simpson indexes, a higher value indicates greater the bacterial community diversity. As shown in Figures 3C,D, the calculated value of the Shannon and Simpson indexes for the *Hp* positive gastric antrum ulcer was significantly lower than that of the *Hp* positive gastric duodenal ulcer (*p* < 0.01, *T*-test), indicating a lower bacterial community diversity.

**Figure 4:**
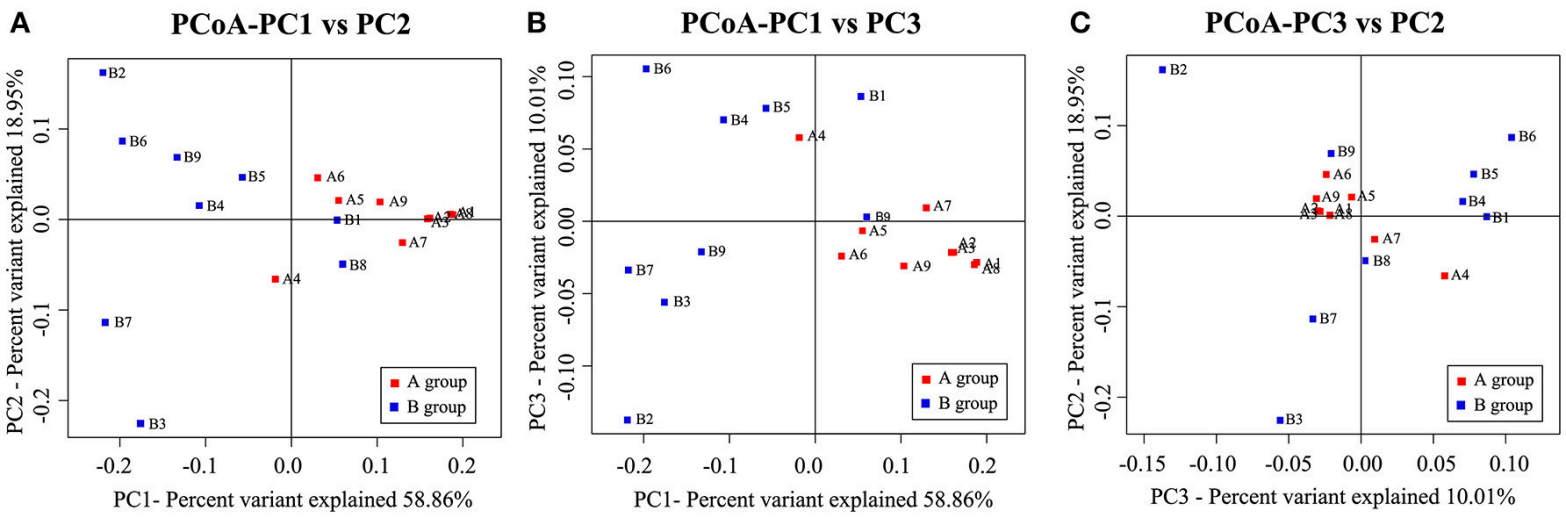
Principal components analysis (PCoA) for bacterial community β-diversity in the *Hp* positive gastric antrum and duodenal ulcer samples. **(A)** PCoA using PC1 and PC2 as main axes; **(B)** PCoA using PC1 and PC3 as main axes; **(C)** PCoA using PC3 and PC2 as main axes.

We then performed clustering based on an unweighted distance matrix and a principal component analysis to characterize the β-diversity for both *Hp* positive gastric ulcer types, which compared the bacterial community difference between every two samples. As shown in Figure 4, the main axis 1 (PC1), 2 (PC2), and 3 (PC3) can explain 58.86, 18.95, and 10.01% of the variation, respectively. It is noteworthy that the samples of the *Hp* positive gastric antrum ulcer were mainly clustered in the positive-value quadrant, and their distribution was relatively concentrated. The samples of the *Hp* positive gastric duodenal ulcer were mainly clustered in the negative-value quadrant, and their distribution was relatively dispersed. This indicated that the intra-sample similarity is higher for the *Hp* positive gastric duodenal ulcer than for the *Hp* positive gastric antrum ulcer.

## Discussion

Previously, it has been well acknowledged that an acidic environment in the stomach and other antibacterial factors are not suitable for bacterial colonization; thus, the stomach should be in a relatively sterile state (1981). With the development of molecular biology and sequencing technology, *Hp* was first discovered in the stomach, and then other microorganisms were also identified (1981; Wang and Yang, 2014). The close relationship between an *Hp* infection and peptic ulcers has long been known; for example, in Japan, 94% of patients with gastric ulcer and 98% of patients with duodenal ulcer have an associated *Hp* infection (Alam et al., 2014). In Middle, duodenal ulcers occur in the duodenal bulb, and gastric ulcers occur in the antrum and other parts of the stomach. Even though *Hp* is the leading cause of ulcers, few studies found differences in the microorganism populations between the two ulcer types. In this study, we compared the mucosal flora structure in 18 cases of *Hp* positive gastric antral ulcer and duodenal ulcer.

Comparing bacteria at the phylum level, we found that Mycobacterium, Bacteroides, and *Firmicutes* were predominant in both ulcer types, which was consistent with the results reported by Bik et al. (Bik et al., 2006). In a genus level comparison, we also identified *Prevotella, Neisseria*, and *Streptococcus* in the *Hp* positive mucosal tissues in both ulcer types, despite the predominance of the bacterium *Helicobacter*. These bacteria are mainly found in the mouth and in food, and their presence has been confirmed in the stomach of the *Hp* negative patients in earlier studies (Jiang et al., 1990; Chen, 2004). Using a mass spectrometry biotyping analysis, Hu et al. (2012) found that *Streptococcus* and *Neisseria* were predominant bacterial genera in *Hp* positive gastric mucosal tissues. Using a 16S rDNA high-throughput sequencing analysis (Li et al., 2009; Ahn et al., 2013) also found that *Streptococcus* and *Neisseria* were predominant bacterial genera inout *Hp* negative gastric antral ulcer patients, and their colonization clearly affected that of *Neisseria* and *Streptococcus* as predominantly observed in the stomach of *Hp* negative patients.

However, few literature reports about the duodenal mucosal floral structure exist. Our study shows that in *Hp* positive duodenal ulcer patients, the proportion of *Helicobacter* is significantly lower than that of *Hp* positive gastric antrum ulcer patients (Figure 1C), whereas *Pseudomonas, Neisseria*, and *Streptococcus* accounted for a relatively larger proportion, so *Helicobacter* was not the only predominant genus. In addition, the proportion of *Bacteroides* and *Streptomyces* in patients with duodenal ulcers was significantly higher than that in patients with gastric antrum ulcers; this outcome was also the case for the bacterial genera *Halophilus, Frankesia, Prevobacteria*, and *Porphyromonas*. An explanation might be that the duodenum is part of the small intestine, which is closer to the jejunum and therefore will be affected by the diversity of the intestinal flora. It might also result from the fact that mucosal cells in the duodenum do not secrete gastric acid, and the relatively high pH value is more suitable for the growth of other flora.

With respect to biodiversity, the bacterial diversity in the mucosal tissues of patients with an *Hp* positive duodenal ulcer was significantly higher than that in patients with an *Hp* positive gastric antrum ulcer (Figures 3A–D, 4). The clinical course of gastric ulcers is usually longer than that of duodenal ulcers and has a relatively higher probability of oncogenesis. The relationship between the lower diversity of gastric flora and oncogenesis has been reported (Ahn et al., 2013). Another advantage of floral diversity is that the metabolites are richer, especially the metabolites that are probiotics, which can inhibit the proliferation of the *Hp* to a certain extent Song (2012). Thus, the findings of our study highly suggest that gastric ulcer treatment can be improved by eradicating the *Hp* infection together with probiotic adjuvant therapy to rebuild the diversity of gastric flora and maintain the stability of the stomach micro-ecological environment.

In conclusion, we compared the bacterial community composition and diversity in the mucosal tissues of *Hp* positive duodenal ulcer and gastric antrum ulcer patients using a 16S rRNA sequencing analysis and identified substantial differences. We believe that similar studies involving more patients that use other bacterial detection methods will further elucidate the relationship between the floral structure and different types of peptic ulcers, which will finally benefit clinical therapies.

## Author contributions

XC and ZW conceived and designed the study; CX, QL, and LJ contributed to collected samples; LZ performed the laboratory tests; XC and ZW participated in analyzing data and writing the manuscript. All authors read and approved the final manuscript.

### Conflict of interest statement

The authors declare that the research was conducted in the absence of any commercial or financial relationships that could be construed as a potential conflict of interest.
